# Fabricated Flexible Composite for a UV-LED Color Filter and Anti-Counterfeiting Application of Calcium Molybdate Phosphor Synthesized at Room Temperature

**DOI:** 10.3390/ma15062078

**Published:** 2022-03-11

**Authors:** Jae-Yong Jung

**Affiliations:** Research and Business Development Foundation, Engineering Building, Silla University, Busan 45985, Korea; eayoung21@naver.com; Tel.: +82-51-999-6441

**Keywords:** CaMoO_4_, co-precipitation, phosphors, synthesis

## Abstract

Crystalline CaMoO_4_ and rare-earth-doped CaMoO_4_:RE^3+^ (RE = Tb, Eu) phosphors were synthesized at room temperature using a co-precipitation method. The crystal structure of the synthesized powder was a tetragonal structure with a main diffraction peak (112) phase. When CaMoO_4_ was excited at 295 nm, it showed a central peak of 498 nm and light emission in a wide range of 420 to 700 nm. Rare-earth-ion-doped CaMoO_4_:Tb^3+^ was excited at 288 nm and a green light emission was observed at 544 nm, and CaMoO_4_:Eu^3+^ was excited at 292 nm and a red light emission was observed at 613 nm. To take advantage of the light-emitting characteristics, a flexible composite was manufactured and a color filter that could be used for UV-LEDs was manufactured. In addition, it was suggested that an ink that could be checked only by UV light could be produced and applied to banknotes so as to prevent counterfeiting.

## 1. Introduction

Rare-earth ions doped in a phosphor act as a sensitizer to transfer energy to the host lattice, or as an activator that receives energy and emits light. To apply phosphors to light-emitting devices such as display panels and white-light-emitting diodes, it is necessary to develop host lattice and activator ions with a high luminous efficiency and good color purity [1,2]. Many researchers are developing various synthetic methods, such as sol−gel methods [3], solid-state reactions [4], and solvothermal synthesis [5] approaches, as part of an effort to develop a high-purity phosphor doped with rare-earth ions. Among the work on phosphors with good color purity and high luminous efficiency, research intended to facilitate the use of molybdate phosphors doped with rare-earth ions in various applications, such as light-emitting devices, lasers, scintillation materials, optical fibers, catalysts, and displays, is attracting attention [6,7,8]. Specifically, calcium molybdate (CaMoO_4_) can be used in a wide range of applications due to its stable physicochemical properties and the low absorption critical energy of its scheelite structure [9,10]. Kim et al. produced CaMoO_4_:Eu^3+^ with Na^+^ phosphors with a diameter of 5 nm from a TOA-MoO_4_ complex with calcium, europium, and sodium-oleate (Ca, Eu, and Na-oleate) mixed in toluene using a solvothermal synthesis method, and suggested that the strongest red emission occurred in Ca_0.58_MoO_4_:_0.21_Eu^3+^, with _0.21_Na^+^ nanophosphors synthesized at 120 °C [11]. Wang et al. used a molten salt method at 270 °C to successfully synthesize nanoparticles with a uniform crystal grain size of about 70 nm with an approximately spherical shape. CaMoO_4_, with a peak at 508 nm when irradiated with an excitation wavelength of 356 nm, was synthesized [12]. Lim et al. prepared CaMoO_4_ particles co-doped with erbium and ytterbium ions using a microwave-assisted metathetic method and observed a green light emission signal at 525 nm under excitation at 980 nm [13]. Pereia et al. prepared a precursor using sol−gel heat-treated at 800 °C, synthesized a SrWO_4_ phosphor with Eu^3+^ added thereto, and synthesized a red phosphor with a main peak at 614 nm when excited at 393 nm [14]. Xia et al. synthesized a BaMoO_4_ phosphor doped with Sm^3+^ at 850 °C using a solid-state method, and observed and reported the change in emission intensity according to doping the materials by doping with Li, Na, and K [15]. In addition to the application cases of phosphors by doping rare earth materials with these metal-based materials, a case of applying copper-added Co_3_O_4_ nanocrystals using hydrothermal synthesis to flexible supercapacitors utilizing the conductivity and particle porosity of the material was reported [16]. The studies reported for synthesizing rare-earth-doped CaMoO_4_ phosphors lacked examples of applications and disadvantages such as heat treatment at high temperature, small amount of synthesis, and long synthesis time. In this study, methods for reducing energy use and various use cases were presented by simplifying the synthesis method and eliminating the heat treatment process. Using the co-precipitation method, crystalline CaMoO_4_ was synthesized at room temperature and doped with rare-earth ions to synthesize green and red phosphors. To take advantage of these unique light-emitting properties, a flexible composite was made by mixing it with a polymer (silicone base), after which it was manufactured as a color filter feasible for use in a UV-LED chip. In addition, it was suggested that an ink that reacts only with UV light could be produced and applied to banknotes to discourage counterfeiting.

## 2. Materials and Method

### 2.1. Synthesis of CaMoO_4_ and CaMoO_4_:RE^3+^ via Co-Precipitation at Room Temperature

**Figure 1:**
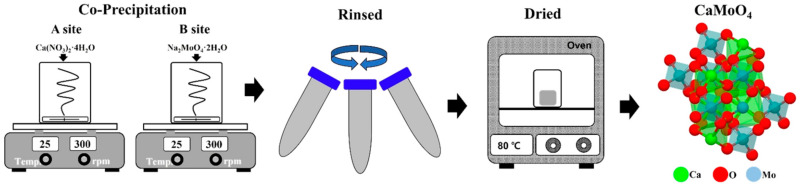
Schematic of the SrMoO_4_ synthesis procedure via co-precipitation at room temperature.

Two beakers were prepared to synthesize crystalline CaMoO_4_ by co-precipitation at room temperature. Here, 1 mmol of calcium nitrate and 100 mL of distilled water were added to beaker “A” and stirred. The same moles of sodium molybdate and distilled water were added to beaker “B”, which was stirred until they were completely dissolved. After 1 h, the two solutions were confirmed to be completely dissolved, and the solution was slowly poured from beaker “B” into beaker “A” and stirred for 3 h. Centrifugation was then conducted to recover the reacted white powder, and the powder was recovered after washing twice to remove the unreacted substances and sodium. The recovered powder was placed in an oven at 80 °C and dried for about 12 h. To use crystalline CaMoO_4_ as a phosphor material, 0.25 mmol of rare-earth ions (terbium (III) nitrate; Tb^3+^, europium (III) nitrate; Eu^3+^) were added to beaker A during the co-precipitation step, and were synthesized using an identical process (Figure 1).

### 2.2. Fabricated of a Flexible Composite

First, 1 wt% of the synthesized CaMoO_4_ and CaMoO_4_:RE^3+^ phosphors were mixed with 5 g of polydimethylsiloxane (PDMS), and were poured into a square mold, put in a vacuum oven at 80 °C, and cured for 2 h to prepare a flexible composite. The prepared composite was photographed for comparison with typical daylight and a UV-lamp, and the color change was observed and photographed by placing it on a UV-LED chip. An anti-counterfeiting ink was prepared by mixing 0.5 wt% of phosphor powder synthesized by doping with rare-earth ions, glycerin (2 g), and ethyl alcohol (4 g). The prepared ink was applied to a bank note with a brush and was examined with the naked eye and under a UV lamp.

### 2.3. Characterization

To confirm the crystal structure of CaMoO_4_ synthesized by the co-precipitation method, it was measured at a diffraction angle (2 θ) of 10 to 70° using an X-ray diffraction (XRD; Rigaku Ultima IV, Akishma-shi, Tokyo, Japan) apparatus at a rate of 2° per minute. The size and surface shape of the particles were analyzed through a field-emission transmission microscope (FE-SEM; SU-8220, Hitachi, Chiyoda-ku Tokyo, Japan). The optical properties were investigated at a photomultiplier voltage of 350 V using a fluorescence photometer (Scinco, FS-2, Seoul, Korea) at room temperature. The chemical bonding state was investigated by X-ray photoelectron spectroscopy (XPS; ESCALAB 250 XI, Waltham, MA, USA) and the actual emission wavelength of the manufactured composite was observed using an LED photometer (Gigahertz Optik, S-BTS256, Tuerkenfeld, Germany).

## 3. Results and Discussion

### 3.1. Structure and Surface Morphology of Crystalline CaMoO_4_

**Figure 2:**
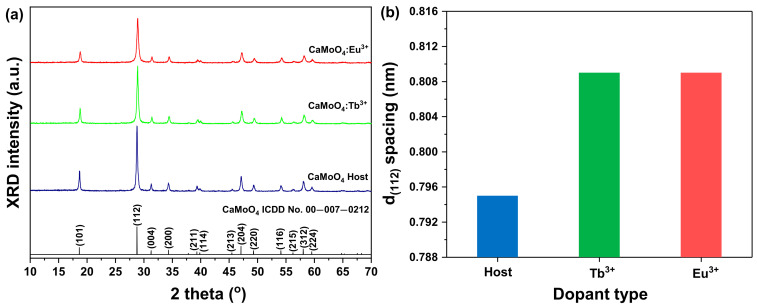
(**a**) XRD patterns and (**b**) d_(112)_ spacing of CaMoO_4_ and CaMoO_4_:RE^3+^.

Figure 2a shows the XRD patterns of the CaMoO_4_ and CaMoO_4_:RE^3+^ (RE = Tb^3+^, Eu^3+^) samples synthesized at room temperature via co-precipitation. MXO_4_ (M = Ca, X = Mo) particles were precipitated―M^2+^cation acted as an electron-to-acceptor (Lewis acid) and reacted with the XO_4_^2−^ anion as an electron-to-donor (Lewis base). The reaction between these two species (M^2+^ ←: XO_4_^2−^) continued to produce bonds. The minimum molecular orbital energy of the Lewis acid interacted with the highest molecular orbital energy of the Lewis base, resulting in the synthesis of MXO_4_ particles [17,18]. For the three samples, peak signals for the (101), (112), (204), (220), and (312) phases were observed, consistent with ICDD card #00-007-0212 and tetragonal (a = 5.2260 Å, b = 5.2260 Å, c = 11.430 Å).

When the doped rare-earth ions were calculated using a single unit cell of CaoMO_4_, the calculation showed that the doped amount was about 1.557 × 10^19^ RE atoms/cm^3^ (RE = Tb^3+^, Eu^3+^; the calculation process is explained in detail in the Supporting Information). When the diffraction angle of the (112) phase, which is the main diffraction peak, was substituted into Bragg’s equation [19] and compared, as shown in Figure 2b, the lattice constant of the sample to which the rare-earth ions were added was increased (CaMoO_4_ = 0.795, CaMoO_4_:Tb^3+^ = 0.809, CaMoO_4_:Eu^3+^ = 0.809). This is thought to be due to the lattice change caused by the doping of rare-earth ions with relatively large ionic radii into the lattice [16]. In addition, the particle size “D” was calculated by substituting the main diffraction angle and full width at half maximum (FWHM) into Scherrer’s equation, as indicated in Equation (1) [19].

(1) D=nλ/Bcosθ

**Figure 3:**
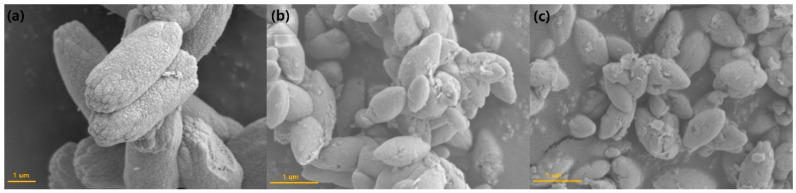
FE-SEM images of (**a**) CaMoO_4_, (**b**) CaMoO_4_:Tb^3+^, and (**c**) CaMoO_4_:Eu^3+^.

Here, *n* is Scherrer’s constant 0.9, *λ* is the X-ray wavelength (0.15406 nm), *θ* is the diffraction angle, and *B* is the FWHM [16]. Accordingly, CaMoO_4_ was 30 nm, and for the Tb^3+^ and Eu^3+^ single-doped samples, the particle sizes were 29 nm and 25 nm, respectively. The FE-SEM images of the crystal grain sizes and surface shapes of the synthesized CaMoO_4_ and CaMoO_4_:RE^3+^ (RE = Tb^3+^, Eu^3+)^ samples are shown in Figure 3. The CaMoO_4_ sample had a cylindrical particle model approximately 3.5 um in size in the longitudinal direction and close to 1.9 um in size in the transverse direction, as particles of about 50 nm in size were aggregated (Figure 3a). In the CaMoO_4_:RE^3+^ sample, particles approximately 47 nm in size were agglomerated and had a grain shape with a length of about 1 μm and width of about 0.5 μm (Figure 3b,c). In the FE-SEM EDS mapping component analysis, components such as Ca, Mo, and O were confirmed, and in the rare-earth-doped specimen, the components of Tb and Eu were also confirmed, as shown in Figure S1. In this work, it was shown that the crystalline CaMoO_4_ and CaMoO_4_:RE^3+^ samples synthesized by the co-precipitation method could be synthesized at room temperature without an additional heat treatment process.

### 3.2. Chemical States and Luminescence Properties of the CaMoO_4_ Phosphor

**Figure 4:**
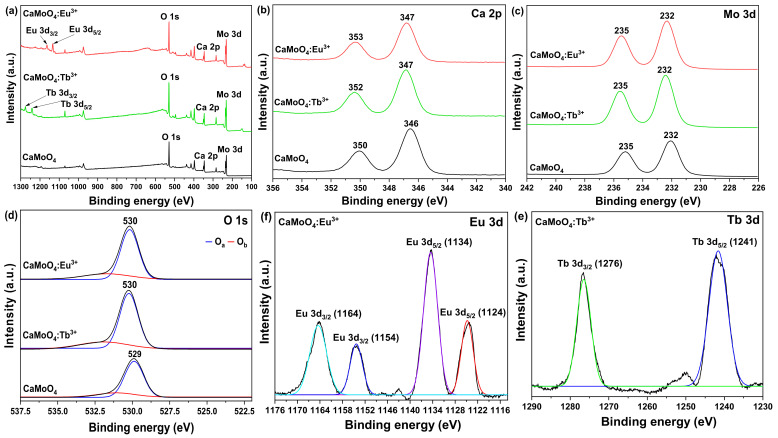
(**a**) XPS survey, (**b**) Ca 2P, (**c**) Mo 3d, (**d**) O 1s, (**e**) Tb 3d, and (**f**) Eu 3d spectra of CaMoO_4_ and CaMoO_4_:RE^3+^.

An XPS analysis was conducted to determine the chemical state of the synthesized CaMoO_4_ and CaMoO_4_:RE^3+^ samples (Figure 4). The XPS peak corresponded to Ca (2p) with binding energy (BE) outcomes of 346.48 eV (2p_3/2_) and 350.08 eV (2p_1/2_). Upon doping with Tb^3+^ (346.98 eV and 351.68 eV) and Eu^3+^ (346.78 eV and 352.78 eV) ions, the binding energy value shifted slightly higher (Figure 4b). From the above results, it is assumed that calcium was stable in the +2 oxidation state in both samples. Figure 4c shows the XPS spectra of Mo in the Mo 3d region of undoped CaMoO4 and rare-earth doped CaMoO_4_ [20]. The peaks around 232 and 235 eV were characteristic of MoO_3_, probably due to Mo^6+^ 3d_5/2_, and 3d_3/2_, respectively [21]. Mo (3d) showed core BE outcomes of 232.08 eV (3d_5/2_) and 235.48 eV (3d_3/2_). Upon doping with rare-earth ions, the binding energy value shifted slightly to the lower energy side (232.05 and 235.38 eV; CaMoO_4_:Tb^3+^, 232.04 and 235.38 eV; CaMoO_4_:Eu^3+^). Based on these results and the BE values, it was concluded that molybdenum was stable in the +6 oxidation state in both undoped and rare-earth-doped CaMoO_4_. Figure 4d presents the O 1s core-level peaks of the CaMoO_4_ and CaMoO_4_:RE^3+^ samples. In this case, the O 1s core-level peak was slightly asymmetric at ~530 eV and fitted two symmetric Gaussian curves, denoted by O_a_ (blue line) and O_b_ (red line) peaks. The O_a_ peak was attributed to the lattice oxygen atoms of CaMoO_4_. There were quite a few reports indicating that the high-energy peak (O_b_) of O 1s was due to hydroxyl or chemisorbed oxygen and organic oxygen on the sample surface, such as CO or COO^−^ [22]. However, the asymmetric feature of the high-energy peak (O_b_) of the O 1s XPS spectrum demonstrated the presence of oxygen vacancies in the sample. Gupta et al. observed that the O_b_ peak developed with an increase in the number of oxygen vacancies [23]. Comparing the integrated area ratio of O_b_/O_a_ in CaMoO_4_ and CaMoO_4_:RE^3+^, it can be seen that the area ratio of the CaMoO_4_:RE^3+^ was higher than that of CaMoO_4_, as well as the oxygen vacancy concentration. This was consistent with the fact that after doping divalent Ca^2+^ sites with trivalent europium ions, the concentration of defects and oxygen vacancies increased [23]. The XPS spectrum outcomes of the rare-earth 3d levels of the CaMoO_4_:Tb^3+^ and CaMoO_4_:Eu^3+^ samples are correspondingly shown in Figure 4e,f. Two peaks in the Tb^3+^ 3d region are clearly shown in the spectrum of two peaks. The peak 1241 eV in both samples corresponds to Tb^3+^ 3d_5/2_, the peak at 1276 eV (Figure 4e). There are four peaks in the Eu^3+^ 3d region. The peaks 1124 and 1134 eV in the corresponding samples correspond to Eu^3+^ 3d_5/2_, whereas the peaks at 1154 and 1164 eV monitored (Figure 4f). The BE values for Tb^3+^ and Eu^3+^ 3d_5/2_ were consistent with those reported for rare-earth coordinated ions, indicating that the oxidation state of rare-earth ions was +3 in both cases [24,25].

**Figure 5:**
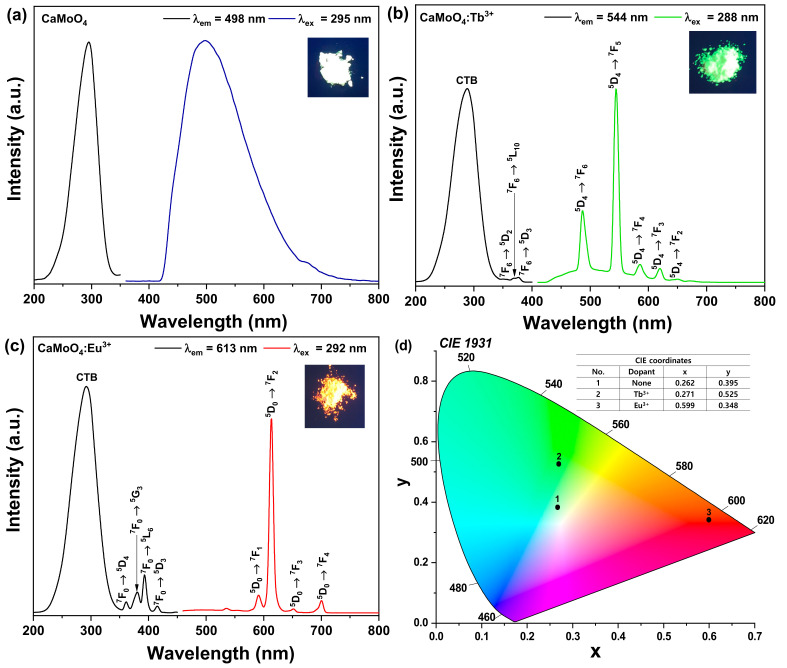
PLE and PL spectra of (**a**) CaMoO4, (**b**) CaMoO4:Tb3+, (**c**) CaMoO4:Eu3+, and (**d**) CIE coordination.

The photoluminescence excitation (PLE) and photoluminescence (PL) spectra of the CaMoO_4_ phosphor are shown in Figure 5a. The PLE spectrum (black line) was monitored at an emission wavelength at 498 nm. This spectrum showed a broad peak centered at 295 nm, which arose due to the Mo–O charge-transfer band [26]. The PL spectrum (blue line) was monitored at an excitation wavelength at 295 nm, showing a broad band around 498 nm, attributable to the intrinsic luminescence of the host lattice. Figure 5b shows the PLE spectrum (black line) of the CaMoO_4_:Tb^3+^ phosphor monitored at an emission wavelength of 544 nm. The PLE spectrum of the CaMoO_4_:Tb^3+^ phosphors contained a charge transfer state (CTB) and f−f transitions due to the presence of Tb^3+^ ions [27]. CTB includes a broad band in the wavelength range of 220–320 nm. This band could arise due to the Mo-O and Tb–O charge transfer. The f−f transitions associated with a few weak peaks were located at 350, 369, and 376 nm, which were attributed to the ^7^F_6_ → ^5^D_2_, ^7^F_6_ → ^5^L_10_, and ^7^F_6_ → ^5^D_3_ transitions of the Tb^3+^ ions, respectively. The PL spectrum (green line) was observed for the excitation wavelength of 288 nm, stemming from the Tb–O CTB. The emission spectra contained five peaks centered at 486, 544, 586, 620, and 650 nm wavelengths corresponding to the ^5^D_4_ → ^7^F_6_, ^5^D_4_ → ^7^F_5_, ^5^D_4_ → ^7^F_4_, ^5^D_4_ → ^7^F_3_, and ^5^D_4_ → ^7^F_2_ transitions [28]. These transitions were well known characteristic transitions of Tb3+ ions. Among the above five types of emission spectra, the intensity of the green emission spectrum due to the ^5^D_4_ → ^7^F_5_ (544 nm) magnetic dipole transition was the strongest, and this emission intensity was the strongest due to the ^5^D_4_ → ^7^F_6_ (486 nm) electric dipole transition of the Tb^3+^ ion [29]. It was 2.69 times that of the blue emission intensity. From this result, it can be confirmed that the Tb^3+^ ion located in the host crystal was located at an inversion symmetry site. Figure 5c shows the results of the PLE (black line) and PL (red line) spectra when measured in the CaMoO_4_:Eu^3+^ phosphor powder. For the CaMoO_4_:Eu^3+^ phosphor powder controlled to an emission wavelength of 613 nm, two types of PLE spectra were observed. One type of absorption signal was an absorption spectrum by CTB generated between Mo-O ions with a wide bandgap over the region of 220–320 nm and a peak at 292 nm, and the other type of absorption signal is at 320–450 nm. Observed in the wavelength region, these absorption signals were 4f−4f transition signals of Eu^3+^ ions [30]. The absorption spectra observed at the peaks at 361, 381, 393, and 415 nm corresponded to the ^7^F_0_ → ^5^D_4_, ^7^F_0_ → ^5^G_3_, ^7^F_0_ → ^5^L_6_, and ^7^F_0_ → ^5^D_3_ transition signals of the Eu^3+^ ions located in the host crystal [31]. The emission spectrum of the phosphor was measured by exciting the phosphor powder with a wavelength of 292 nm, showing the strongest absorption intensity. The phosphor powder was composed of an emission spectrum with the strongest emission intensity with a peak at a wavelength of 613 nm, a peak emission spectrum at 590 nm with a relatively weak emission intensity, and an emission spectrum with peaks at 651 nm and 700 nm. Here, 613 nm was the signal induced by the ^5^D_0_ → ^7^F_2_ electric dipole transition, 590 nm was the ^5^D_0_ → ^7^F_1_ magnetic dipole transition, and the two red emission spectra (651 and 700 nm) were the ^5^D_0_ → ^7^F_3_ and ^5^D_0_ → ^7^F_4_ electric dipole signals [32]. In this experiment, because the intensity of the red emission (613 nm) spectrum stemming from the ^5^D_0_ → ^7^F_2_ electric dipole transition was 10.9 times greater than the intensity of the orange emission (590 nm) from the ^5^D_0_ → ^7^F_1_ magnetic dipole transition, Eu^3+^ ions in the host lattice were found to be located at the sites of inversion symmetry, not inversion symmetry [33]. Color coordinates were an important factor when evaluating the performance of a phosphor, and a proper understanding of color purity is important when applying these phosphors in the lighting industry. Figure 5d shows the CIE (Commission Internationale de L’Eclairage 1931) color coordinates of the CaMoO_4_ phosphor powder doped with different activator ions. The CIE color coordinates of the phosphor powder were investigated based on the data of the emission spectrum. The numbers shown in Figure 5d indicate the color coordinates of the activator ions No. 1 CaMoO_4_ (0.262, 0.395), No. 2 CaMoO_4_:Tb^3+^ (0.271, 0.525), and No. 3 CaMoO_4_:Eu^3+^ (0.599, 0.348) phosphors.

**Figure 6:**
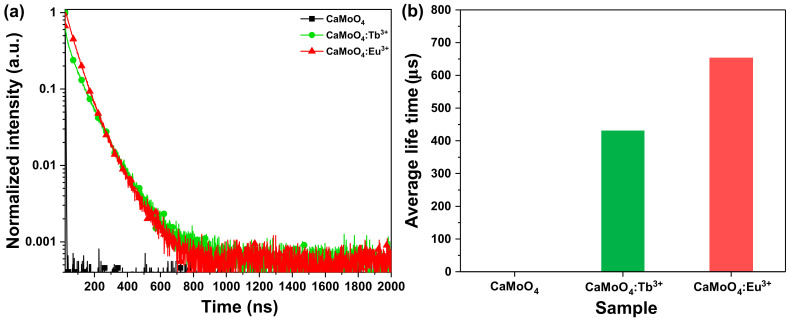
(**a**) Time-resolved PL and (**b**) average decay time.

In addition, we measured the time-resolved photoluminescence (TRPL) for the luminescence dynamics of CaMoO_4_ and CaMoO_4_:RE^3+^, as shown in Figure 6a. After an analysis of the TRPL [34,35], the excitation wavelength was found to be 315 nm. The wavelengths used for the analysis were those of CaMoO_4_, CaMoO_4_:Tb^3+^, and CaMoO_4_:Eu^3+^ emitted at 498 nm, 544 nm, and 613 nm, respectively. Figure 6b shows that the average decay time (τ_avg_) elapsed slowly. It is believed that the light energy received from the host is transferred to the doped rare-earth ions as an activator and that the light emission lifetime of the rare-earth-doped sample is longer than that of the undoped sample due to the mechanism used by the phosphor to emit light (CaMoO_4_ = Non, CaMoO_4_:Tb^3+^ = 431 μs, CaMoO_4_:Eu^3+^ = 654 μs). Zhu et al. observed changes in properties according to the changes in structure, surface shape, and pores according to the doping amount of the carbon material when manufacturing a composite in which a carbon material and a conductive material were mixed. It was announced that the characteristics of a supercapacitor were strengthened by utilizing the characteristics changed by the doping material [36]. In this study, it was found that the structure, particle size, and surface shape of CaMoO_4_ were changed by the addition of rare earth, and the luminescence properties were strengthened. Table S1 compares the properties of CaMoO4 phosphors doped with rare earths synthesized by other methods. It can be seen that the synthesis method presented in this work is easy and can be synthesized at room temperature without additional heat treatment, and the luminescence properties are strong.

### 3.3. Flexible Composite with a UV-LED Color Filter Applied for Anti-Counterfeiting

**Figure 7:**
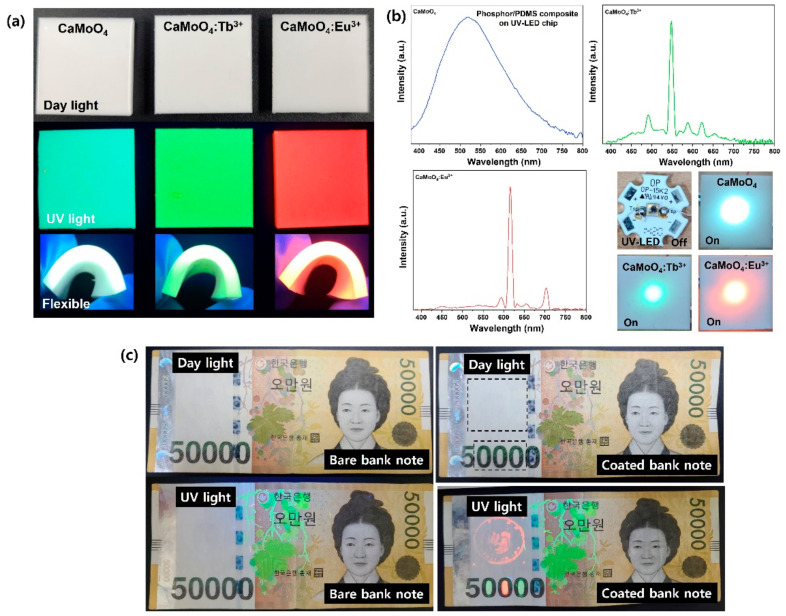
(**a**) Phosphors-PDMS composite under daylight and UV light. (**b**) Composite on UV-LED chip and a detected LED photometer. (**c**) Anti-counterfeiting ink painted on a banknote.

The composite produced by mixing the synthesized phosphor and PDMS was difficult to identify with the naked eye, as all three samples (CaMoO_4_, CaMoO_4_:Tb^3+^, and CaMoO_4_:Eu^3+^) were white squares under typical everyday lighting. However, in the UV lamp, it was possible to distinguish between blue, green, and red by realizing the unique color of the phosphor, and it showed flexibility by being easily bent with a finger (Figure 7a). In addition, the same composite was placed on the UV-LED chip and power was supplied. As in the case of a UV lamp, it showed the unique emission color of the phosphor, and when the actual emission wavelength was measured using an LED photometer, the same result found with the previous PL spectrum was shown here as well (Figure 7b). Ink made of phosphor powder synthesized by doping with rare-earth ions (CaMoO_4_:Tb^3+^ and CaMoO_4_:Eu^3+^) was applied to a bank note with a brush. In daylight, it could not be found with the naked eye, though green and red luminescence could be confirmed with a UV lamp (Figure 7c). This suggests that the flexible composite produced in this study could be used as a UV-LED color filter and that the ink produced using a phosphor could be applied to help prevent counterfeiting.

## 4. Conclusions

At room temperature, crystalline CaMoO_4_ and rare-earth-ion-doped CaMoO_4_:Tb^3+^ and CaMoO_4_:Eu^3+^ powders were synthesized via a co-precipitation method. The synthesized powder had a tetragonal structure with the (112) phase as the main peak, according to the XRD analysis. As the rare-earth ions were doped, a change in the lattice constant was observed, and calculations showed that the doped rare-earth ion was doped with about 1.557 × 10^19^ RE atoms/cm^3^ in the host CaMoO_4_ lattice. When the powder synthesized by FE-SEM was observed, about 50 nm-sized particles of CaMoO_4_ were agglomerated to form particles approximately 3 μm in size. In the XPS analysis, changes in the binding energy of Ca 2p, Mo 3d, and O 1s were observed as the rare-earth ions were added, and the binding energy peaks of Tb 3d and Eu 3d were observed to confirm that rare-earth ions were doped. The synthesized powder had a tetragonal structure with the main diffraction peak in the (112) phase. When CaMoO_4_ was excited at 295 nm, it showed a central peak of 498 nm and light emission in a wide range of 420 to 700 nm. Rare-earth-ion-doped CaMoO_4_:Tb^3+^ was excited at 288 nm and green light emission was observed at 544 nm, and CaMoO_4_:Eu^3+^ was excited at 292 nm and red light emission was observed at 613 nm. To take advantage of the light-emitting characteristics, a flexible composite was manufactured and a color filter that can be used for UV-LEDs was manufactured. In addition, it was suggested that an ink that could be checked only by UV light could be produced and applied to banknotes so as to prevent counterfeiting.

## Data Availability

The data presented in this study are available upon request from the corresponding author.

## Supplementary Materials

The following supporting information can be downloaded at: https://www.mdpi.com/article/10.3390/ma15062078/s1. Figure S1: SEM-EDS mapping data of CaMoO_4_:Tb^3+^ (up) and CaMoO_4_:Eu^3+^ (down); Table S1: The comparison among similar literature.
