# Supplementary material for: Fabricated Flexible Composite for a UV-LED Color Filter and Anti-Counterfeiting Application of Calcium Molybdate Phosphor Synthesized at Room Temperature
Source: Materials (Basel). 2022 Mar 11;15(6):2078. doi: 10.3390/ma15062078 (PMC8949206; doi:10.3390/ma15062078)
Supplement: Supplementary file 1 [file materials-15-02078-s001.zip › materials-1624207-supplementary.pdf]

materials MDPI

Supplementary

Fabricated Flexible Composite for a UV-LED Color Filter and Anti-Counterfeiting Application of Calcium Molybdate Phosphor Synthesized at Room Temperature

Jae-Yong Jung *

Research and Business Development Foundation, Engineering Building, Silla University, Busan 45985, Korea; eayoung21@naver.com; Tel.: +82-51-999-6441

Calculation of Doped Rare Earths in Lattice

The known lattice parameters of $CaMoO_4$: a = 0.522nm, b = 0.522nm, c = 1.143nm

The volume of a single unit cell(v): v = a × b × c = 0.3114 nm^3 = 31.14 × 10^{-23} cm^3

The doping level of RE^{3+} in $CaMoO_4$: <0.5 mol% = 5 x 10^{-3} RE atoms per 1 RE unit cell

Then, the doping concentration of RE in $CaMoO_4$ is

$$c = \frac{5 \times 10^{-3}\ RE\ atoms}{31.14 \times 10^{-23}\ cm^3} = 1.557 \times 10^{19}\ RE\ atoms/cm^3$$

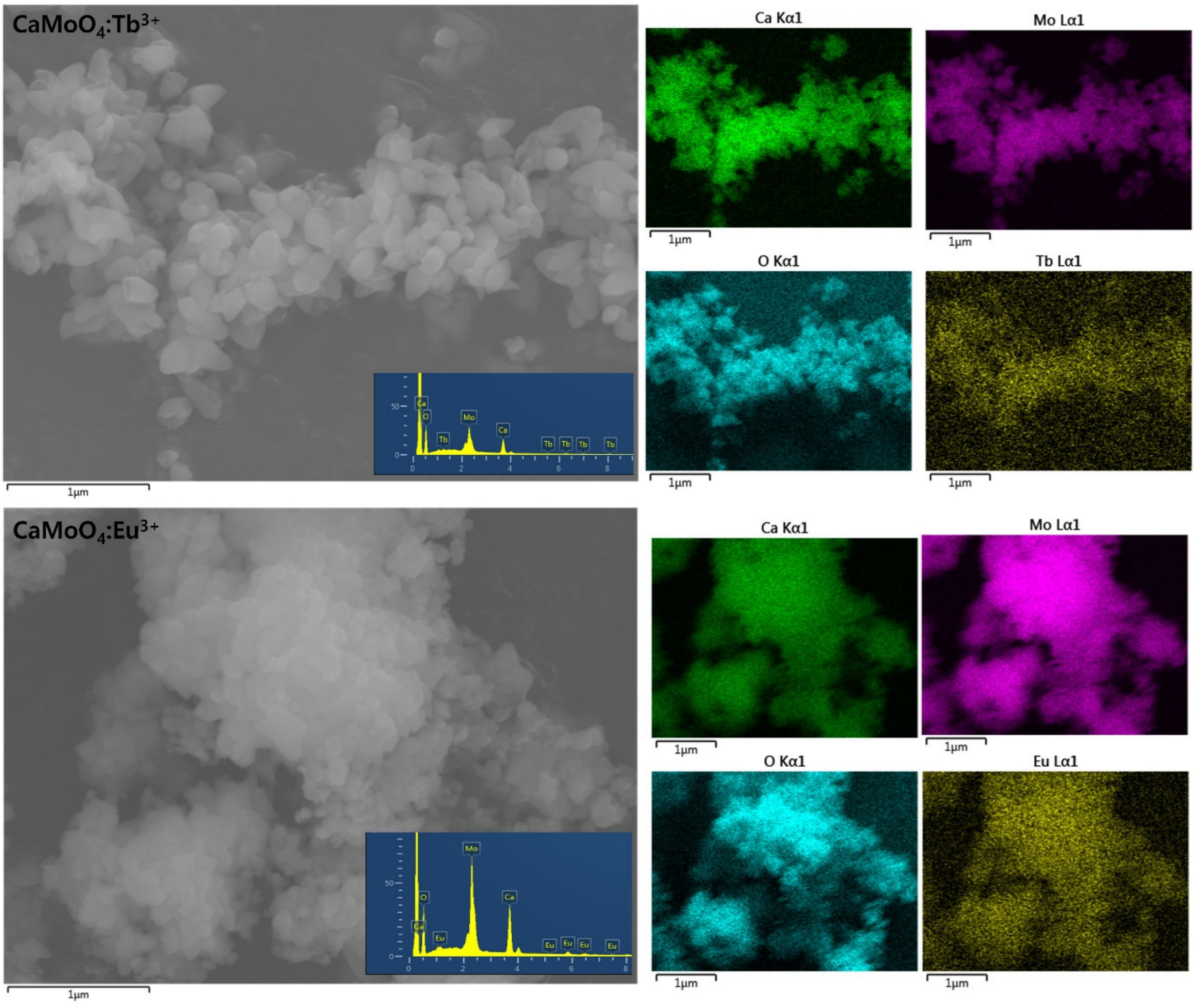

Figure S1. SEM-EDS mapping data of $CaMoO_4:Tb^{3+}$ (up) and $CaMoO_4:Eu^{3+}$ (down).

Table S1. The comparison among similar literature.

No.1	Method	Working Temp.	Working Time	Dopant	Color	Lifetime
[1]	hydrothermal	180 ℃	1 h	Tb, Sm	green, red	0.52, 0.69 μs
[2]	precipitation	120/900 ℃	20/5 h	Eu	red	558 μs
[3]	co-deposition	50/800 ℃	2/6 h	Eu	red	0.637 μs
[4]	solid-state	800 ℃	5 h	Dy	greenish yellow	0.227 μs
[5]	sol-gel	50/700 ℃	2/3 h	Eu	red	20 μs
This work	co-precipitation	25 ℃	1 h	Tb, Eu	green, red	431, 654 μs

References

1. Verma, A.; Sharma, S.K. Rare-earth doped/codoped $CaMoO_4$ phosphors: A candidate for solar spectrum conversion. *Solid state sciences* **2019**, *96*, 105945, DOI 10.1016/j.solidstatesciences.2019.105945.
2. Becerro, A.I.; Allix, M.; Laguna, M.; González-Mancebo, D.; Genevois, C.; Caballero, A.; Lozano, G.; Núñez, N.; Ocaña, M. Revealing the substitution mechanism in Eu^{3+}:$CaMoO_4$ and Eu^{3+} ,Na^{+} :$CaMoO_4$ phosphors. *Journal of materials chemistry. C, Materials for optical and electronic devices* **2018**, *6*, 12830-12840, DOI 10.1039/c8tc04595j.
3. Xie, Y.; Ma, S.; Wang, Y.; Xu, M.; Lu, C.; Xiao, L.; Deng, S. Controlled synthesis and luminescence properties of $CaMoO_4$:Eu^{3+} microcrystals. *Optical materials* **2018**, *77*, 13-18, DOI 10.1016/j.optmat.2018.01.015.
4. Luewarasirikul, N.; Kaewkhao, J. Light-emitting $CaMoO_4$:Dy^{3+} phosphors for photonic materials: Synthesis and luminescence properties. *THE SECOND MATERIALS RESEARCH SOCIETY OF THAILAND INTERNATIONAL CONFERENCE* **2020**, *2279*, DOI 10.1063/5.0023233.
5. Wu, H.; Hu, Y.; Zhang, W.; Kang, F.; Li, N.; Ju, G. Sol–gel synthesis of Eu^{3+} incorporated $CaMoO_4$: the enhanced luminescence performance. *J Sol-Gel Sci Technol* **2012**, *62*, 227-233, DOI 10.1007/s10971-012-2716-8.
